# A systematic review of comparative time to all cause discontinuation of antipsychotic medications in first episode psychosis

**DOI:** 10.1192/bjo.2021.642

**Published:** 2021-06-18

**Authors:** Alice Brooke, Richard Whale, Mihaela Bucur

**Affiliations:** Brighton and Sussex Medical School, Univeristy of Sussex, Sussex Partnership NHS Foundation Trust (SPFT)

## Abstract

**Aims:**

Prompt treatment with medication and intensive psychosocial support are interventions that have been shown to improve function and prognosis in patients with First Episode Psychosis. NICE guidelines state that patients with a first episode of Psychosis should remain on an antipsychotic medication for 1–2 years to reduce risk of relapse, yet most patients stop long before 2 years. This systematic review explores the comparative time to all cause discontinuation of antipsychotics, often used as a marker of real-world treatment effectiveness, in First Episode Psychosis patients.

**Method:**

A literature search was performed across multiple healthcare databases from 1980 to present day in the English Language. Inclusion criteria covered patients with a First Episode of Psychosis aged 14 years and over, and studies that were randomised controlled trials or observational in nature. The primary outcome measure was time to discontinuation of antipsychotic medication. Bias was assessed using the GRADE approach.

**Result:**

11 studies and 3840 patients were included in the review. Seven studies were randomised clinical trials; three were blinded, and four open-label. The remaining four were observational studies. All but one of the studies had a minimum follow-up period of one year (with a maximum of three years). Due to significant methodological heterogeneity across studies, it was not possible to perform meta-analysis. Narrative analysis of the results showed that Olanzapine performed ranked best, and wasbeing taken for the longest time period by patients, followed by Risperidone.

**Conclusion:**

Multiple reviews exist on the efficacy of antipsychotics in First Episode Psychosis, but this is the first one to focus on time to discontinuation as a distinct outcome measure. The review encompasses a large sample size across North America, Eastern Asia and Europe. The interaction of time to discontinuation of antipsychotics with associated symptom levels and medication doses remains an area for further research. The review highlighted the significant differences in statistical methodology across studies in this emerging field, and the need for standardisation in ongoing research. Whilst effectiveness may therefore be greatest for olanzapine, this is outweighed in current guidance by its least favourable metabolic adverse effects profile.

